# Supplementary material for: Conformational dynamics of the Beta and Kappa SARS-CoV-2 spike proteins and their complexes with ACE2 receptor revealed by cryo-EM
Source: Nat Commun. 2021 Dec 20;12:7345. doi: 10.1038/s41467-021-27350-0 (PMC8688474; doi:10.1038/s41467-021-27350-0)
Supplement: Supplementary file 1 — Supplementary Information [file 41467_2021_27350_MOESM1_ESM.pdf]

Supplementary information

Conformational dynamics of the Beta and Kappa SARS-CoV-2 spike proteins and their complexes with ACE2 receptor revealed by cryo-EM

Yifan Wang[1,2,#], Cong Xu[1,#], Yanxing Wang[1,#], Qin Hong[1, 2,#], Chao Zhang[3,#], Zuyang Li[1,2], Shiqi Xu[3], Qinyu Zuo[1], Caixuan Liu[1,2], Zhong Huang[3,*], Yao Cong[1,2,*]

[1] State Key Laboratory of Molecular Biology, National Center for Protein Science Shanghai, Shanghai Institute of Biochemistry and Cell Biology, Center for Excellence in Molecular Cell Science, Chinese Academy of Sciences, Shanghai 200031, China.

[2] University of Chinese Academy of Sciences, Beijing 100049, China.

[3] CAS Key Laboratory of Molecular Virology and Immunology, Institut Pasteur of Shanghai, Chinese Academy of Sciences, University of Chinese Academy of Sciences, Shanghai 200031, China.

[#] These authors contributed equally to this work.

*To whom correspondence may be addressed. Email: cong@sibcb.ac.cn, huangzhong@ips.ac.cn.

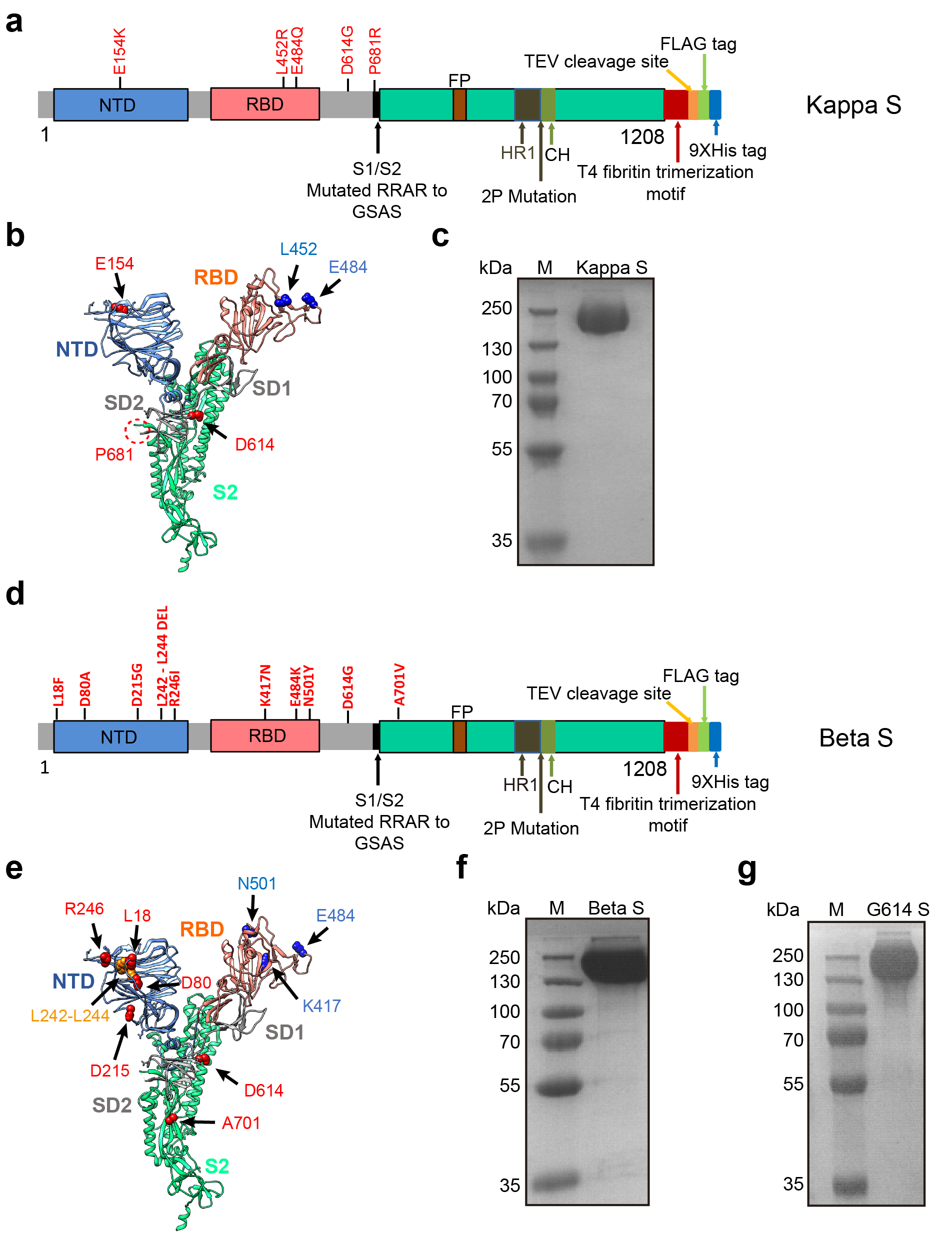

Supplementary Figure 1. Purification of Kappa and Beta S protein. a Schematic diagram of Kappa S organization in this study. Positions of all mutations are shown in red. S1/S2: S1/S2 protease cleavage site, NTD: N-terminal domain, RBD: receptor-binding domain, FP: fusion peptide, HR1: heptad repeat 1, and CH: central helix. **b** Model of SARS-CoV-2 S protomer with mutations of Kappa S shown as sphere. SD1: subdomain 1, SD2: subdomain 2. **c** SDS-PAGE analysis of the purified Kappa S protein. Lane M, protein marker. Representative images of two independent experiments are shown. **d** Schematic diagram of Beta S organization in this study. Positions of all mutations are shown in red. **e** Model of SARS-CoV-2 S protomer with mutations of Beta S shown as sphere. **f-g** SDS-PAGE analysis of the purified Beta S protein (f) and G614 S protein (g). Lane M, protein marker. Representative images of two independent experiments are shown.

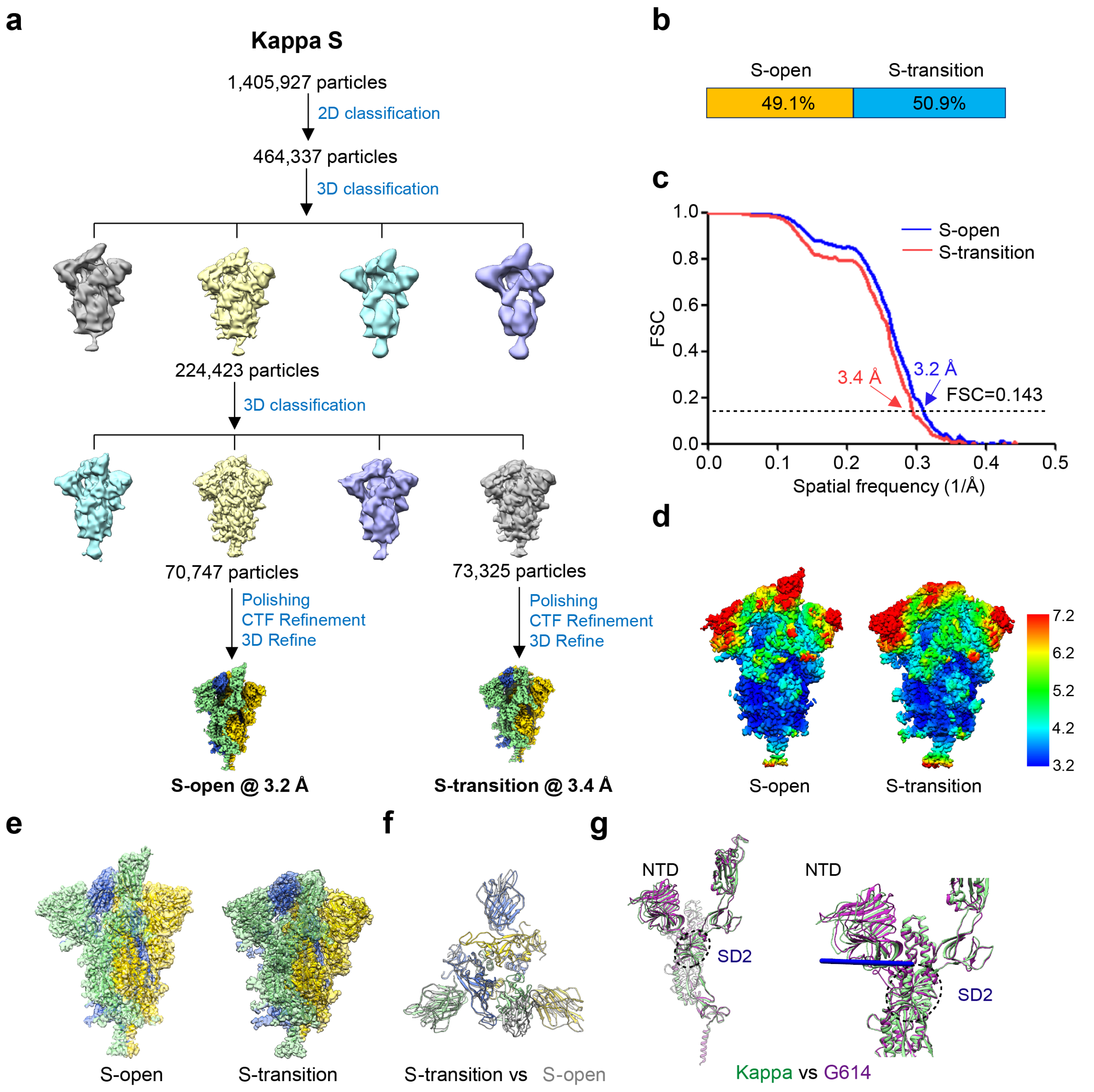

Supplementary Figure 2. Cryo-EM analysis of the Kappa S trimer. a Data processing workflow for structure determination. **b** Population distribution of the Kappa S-open and S-transition. **c** Resolution assessment of Kappa S-open and S-transition maps by Fourier shell correlation (FSC) at 0.143 criterion. **d** Local resolution evaluation of the Kappa S-open and S-transition maps. **e** Model-map fitting of the Kappa S-open and S-transition states. **f** Top view of overlaid Kappa S-transition (in color) and S-open (dark gray) structures. **g** Protomer 1 from the overlaid structures of Kappa S-open (in green) and G614 (PDB: 7KRR, purple), and zoomed-in view of the NTD-SD2 region, with the NTD rotation hinge/axis, located around the region spatially between NTD and SD2, indicated by a blue rod.

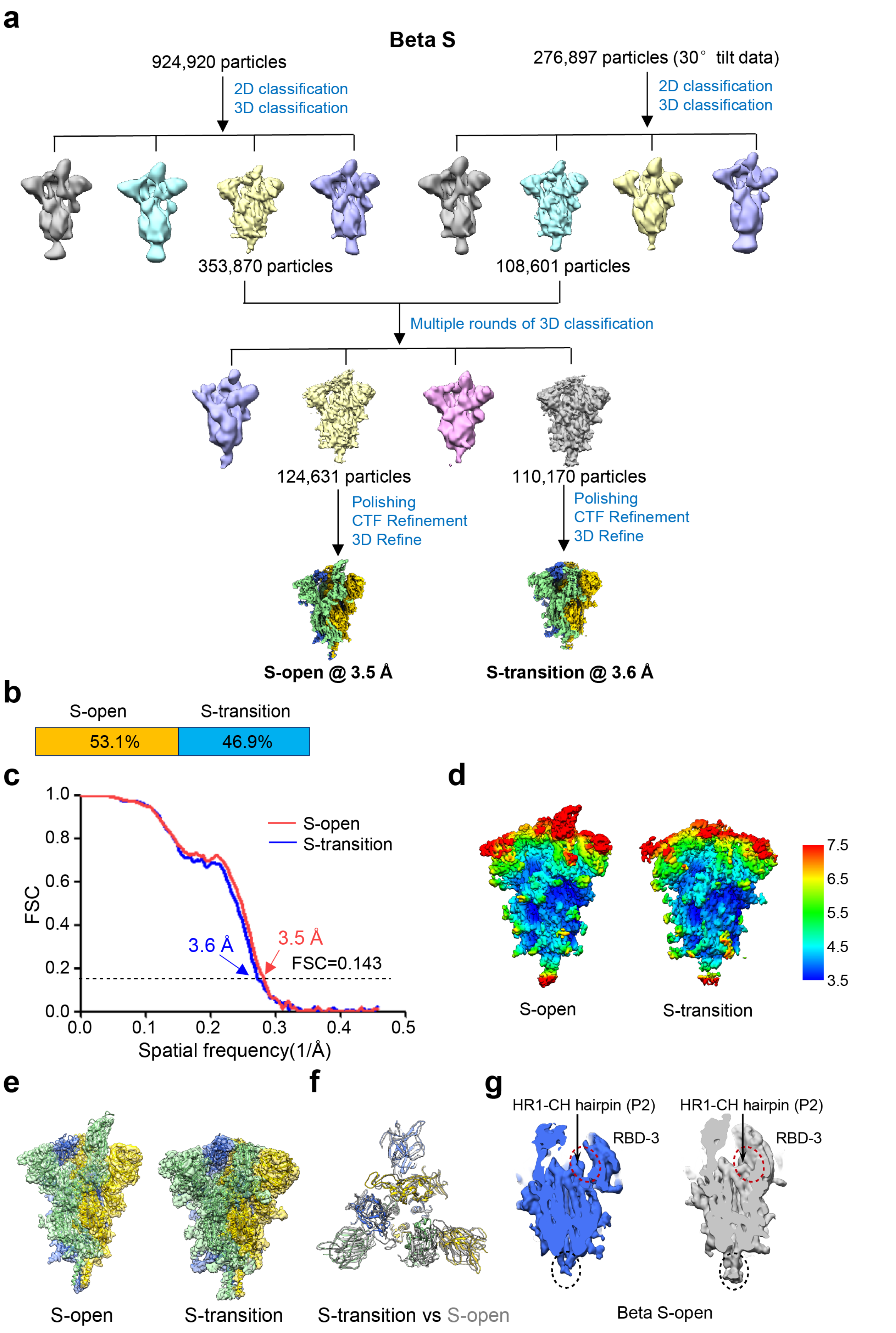

Supplementary Figure 3. Cryo-EM analysis of the Beta S trimer. a Data processing workflow for structure determination. **b** Population distribution of the Beta S-open and

S-transition. **c** Resolution assessment of the Beta S-open and S-transition maps by FSC at 0.143 criterion. **d** Local resolution evaluation of the Beta S-open and S-transition maps. **e** Model-map fitting of Beta S-open and S-transition structures. **f** Top view of the overlaid Beta S-transition (in color) and S-open (dark gray) structures. **g** Central slice view of the 3DVA motion of Beta S-open, displayed in two extreme maps (in royal blue and grey, respectively) in the motion, with key structural elements labeled. S1 subunit RBD-3 movement could be propagated to the central S2 helix bundle of the neighboring protomer 2 through contacting the HR1-CH hairpin.

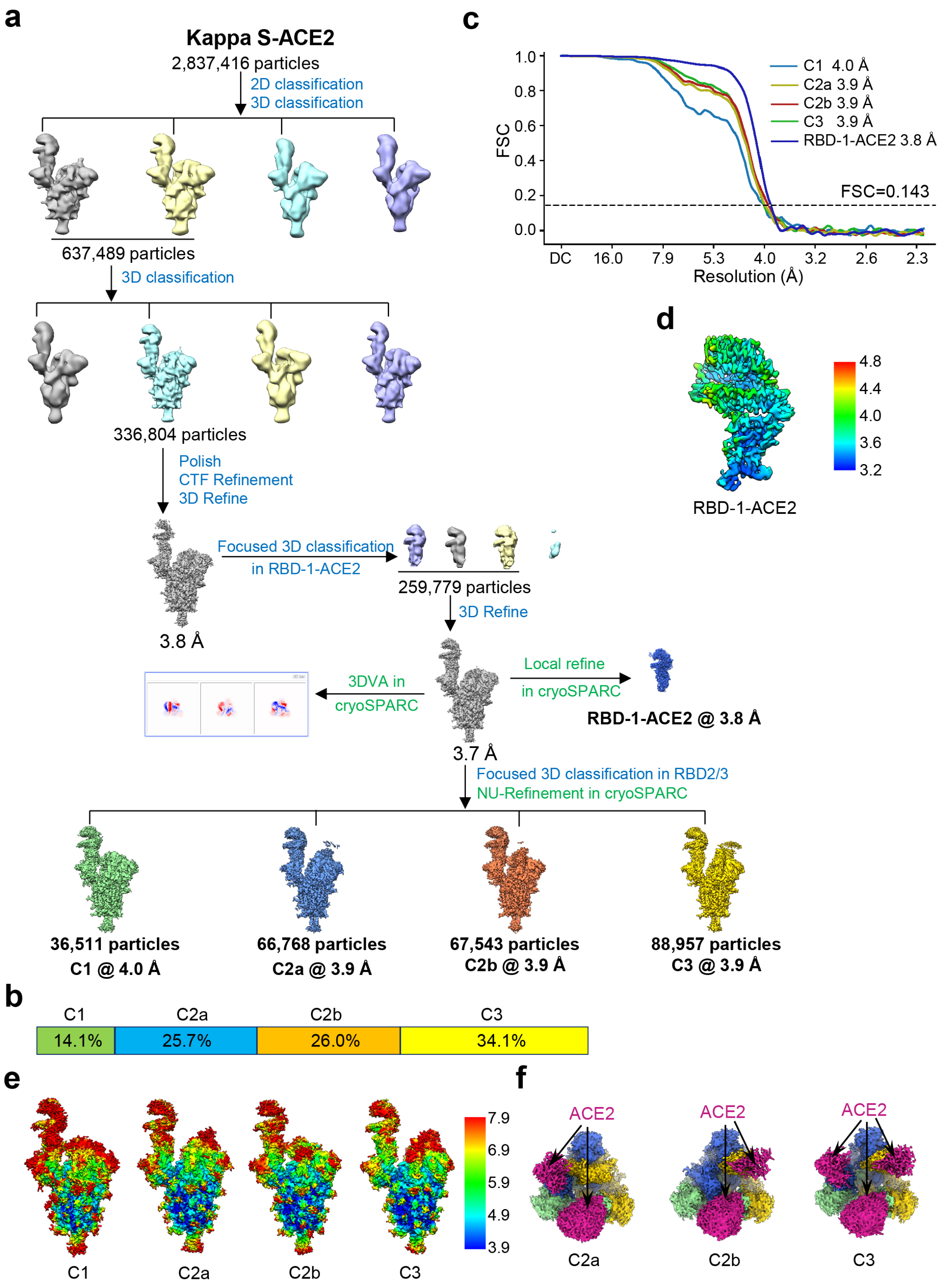

Supplementary Figure 4. Cryo-EM analysis of the Kappa S-ACE2 complex. **a** Cryo-EM data processing procedure for Kappa S-ACE2 complex. **b** Population distribution of the Kappa S-ACE2 conformers. **c** Resolution assessment of the cryo-EM maps by FSC at 0.143 criterion. **d-e** Local resolution evaluation of the RBD-1-ACE2 (d) and S-ACE2 cryo-EM maps (e). **f** Lower threshold rendering of the Kappa S-ACE2-C2a/-C2b/-C3 maps showing ACE2 density (violet red) also associated with RBD-2/3.

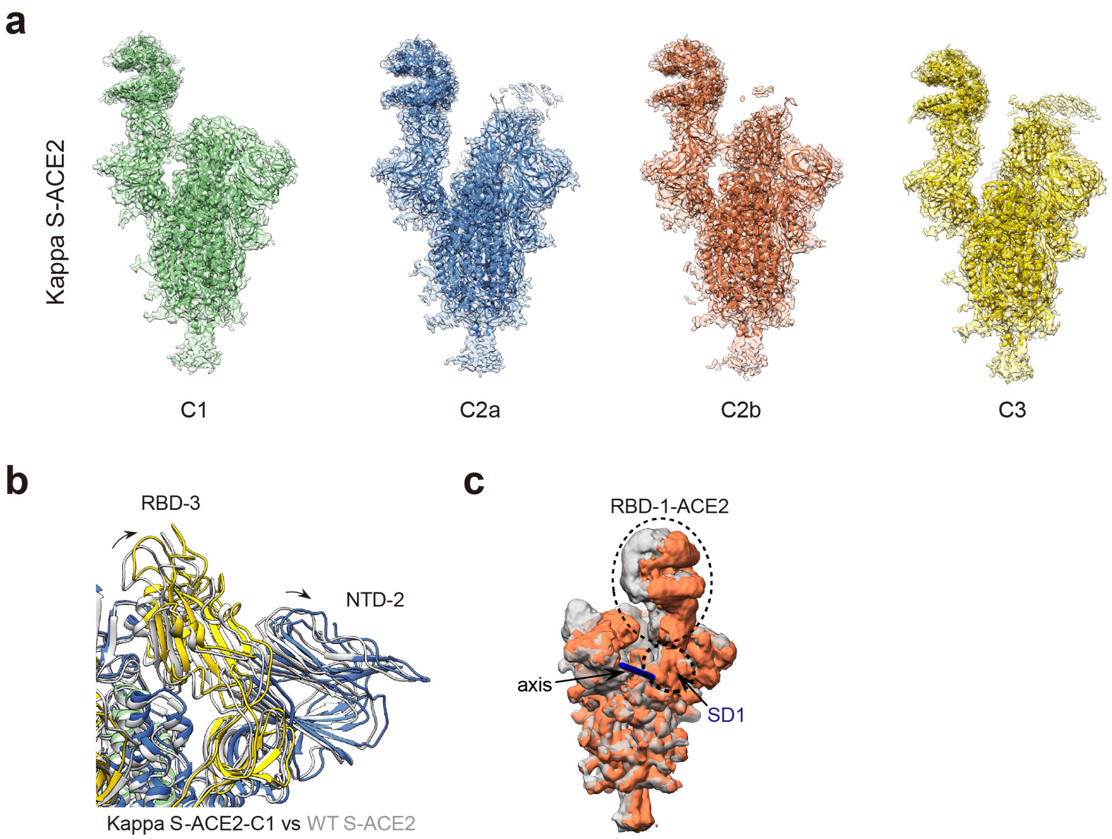

Supplementary Figure 5. Model-map fitting of the Kappa S-ACE2 conformers. a Model-map fitting for the four states of the Kappa S-ACE2 complex. **b** Structural comparasion between the Kappa S-ACE2-C1 (in color) and the WT S-ACE2 (PDB:7DF4, gray), showing movements in RBD-3 and NTD-2. **c** The 3DVA mode 1 motion of Kappa S-ACE2 displayed in maps, showing two extreme conditions in the motion, with the rotation hinge/axis for RBD, around the lower part of SD1, labeled as a blue rod.

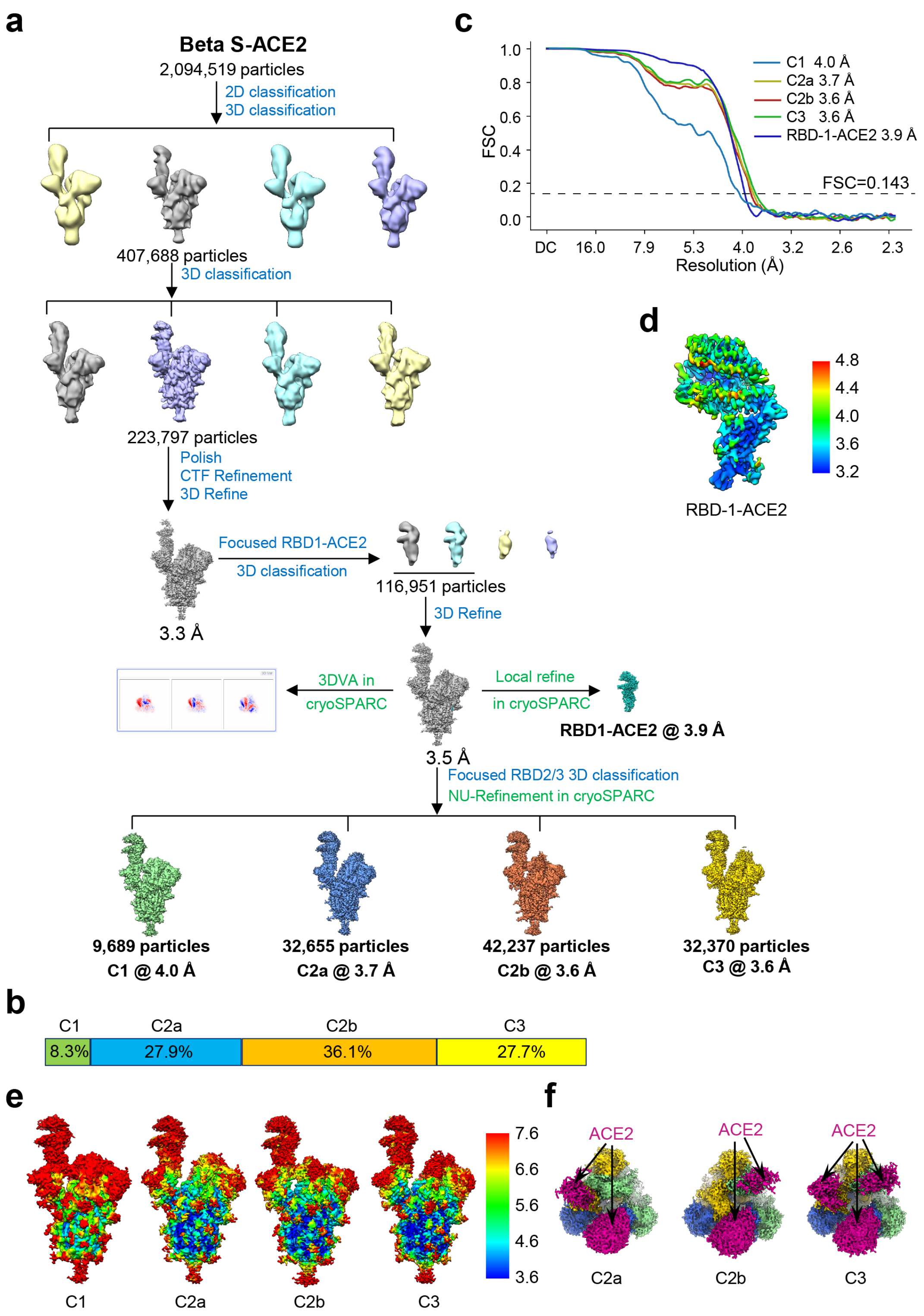

Supplementary Figure 6. Cryo-EM analysis of the Beta S-ACE2 complex. a Cryo-EM data processing procedure for Beta S-ACE2 complex. **b** Population distribution of the Beta S-ACE2 conformers. **c** Resolution assessment of the Beta S-ACE2 cryo-EM maps by FSC at 0.143 criterion. **d-e** Local resolution evaluation of the Beta RBD-1-ACE2 (d) and S-ACE2 cryo-EM maps (e). **f** Lower threshold rendering of the Beta S-ACE2-C2a/-C2b/-C3 maps showing ACE2 density (violet red) also associated with RBD-2/3.

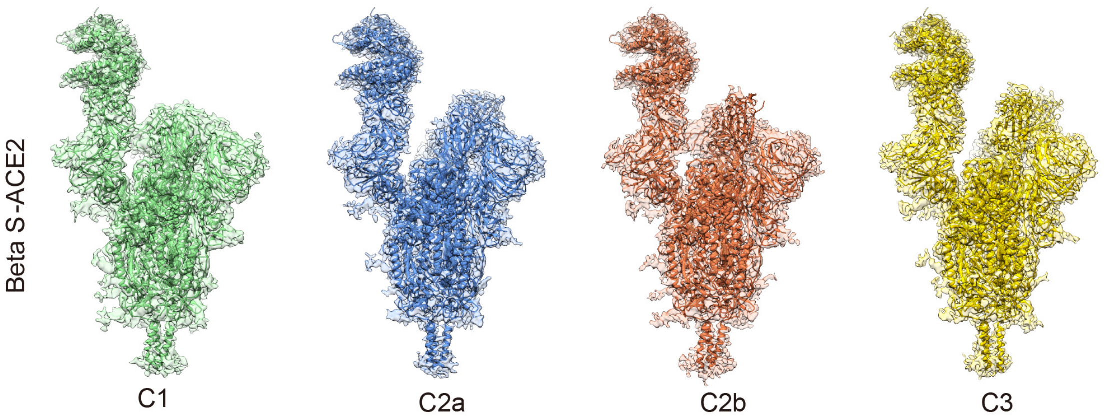

Supplementary Figure 7. Model-map fitting of the Beta S-ACE2 conformers.

Supplementary Table 1. Cryo-EM data collection and refinement statistics for Kappa S and Kappa S-ACE2.

	Kappa S		Kappa S-ACE2				
**Data collection**							
EM equipment	Titan Krios		Titan Krios				
Voltage (kV)	300		300				
Detector	K3		K3				
Pixel size (Å)	1.093		1.093				
Electron dose ($e^-/Å^2$)	50		50				
Exposure time (s)	3		3				
Frames	30		30				
Defocus range (µm)	-0.8 to -2.5		-0.8 to -2.5				
**Reconstruction**							
Softwares	Relion 3.1		Relion 3.1&cryoSPARC				
Structures	S-open	S-transition	C1	C2a	C2b	C3	RBD-1-ACE2
Final particles	70,747	73,325	36,511	66,768	67,543	88,957	259,779
Symmetry	C1	C1	C1	C1	C1	C1	C1
Final overall resolution (Å)	3.2	3.4	4.0	3.9	3.9	3.9	3.8
**Atomic modeling**							
Softwares	Rosetta & Phenix & Coot		Rosetta & Phenix & Coot				
Rms deviations							
Bond length (Å)	0.0033	0.0099	0.0036	0.0036	0.0035	0.0036	0.0045
Bond Angle (°)	0.89	1.46	0.87	0.87	0.87	0.87	0.94
Ramachandran plot (%)							
Favored	95.72	96.10	95.84	95.66	95.83	95.80	98.98
Allowed	4.25	3.67	4.11	4.31	4.14	4.20	1.02
Outliers	0.03	0.23	0.05	0.03	0.03	0.00	0.00

Supplementary Table 2. Cryo-EM data collection and refinement statistics for Beta S and Beta S-ACE2.

	Beta S		Beta S-ACE2				
**Data collection**							
EM equipment	Titan Krios		Titan Krios				
Voltage (kV)	300		300				
Detector	K3		K3				
Pixel size (Å)	1.093		1.093				
Electron dose (e^{-}/Å^{2})	50		50				
Exposure time (s)	3		3				
Frames	30		30				
Defocus range (µm)	-0.8 to -2.5		-0.8 to -2.5				
**Reconstruction**							
Softwares	Relion 3.1		Relion 3.1&cryoSPARC				
Structures	S-open	S-transition	C1	C2a	C2b	C3	RBD-1-ACE2
Final particles	124,631	110,170	9,689	32,655	42,237	32,370	116,951
Symmetry	C1	C1	C1	C1	C1	C1	C1
Final overall resolution (Å)	3.5	3.6	4.0	3.7	3.6	3.6	3.9
**Atomic modeling**							
Softwares	Rosetta & Phenix & Coot		Rosetta & Phenix & Coot				
Rms deviations							
Bond length (Å)	0.0033	0.0099	0.0037	0.0034	0.0034	0.0036	0.0042
Bond Angle (°)	0.89	1.46	0.90	0.91	0.91	0.90	0.93
Ramachandran plot (%)							
Favored	95.72	96.10	95.92	96.08	95.92	95.97	98.99
Allowed	4.25	4.89	3.97	3.84	3.97	3.95	0.88
Outliers	0.03	0.11	0.11	0.08	0.11	0.08	0.13

Supplementary Table 3. Kappa S-ACE2 structure revealed RBD-ACE2 interactions.

RBD from SARS-CoV-2 S		ACE2		Interaction	Distance (Å)
Residue	Atom	Residue	Atom		
K417	NZ	D30	OD2	Salt bridge	2.87
Y449	OH	D38	OD1	H-bond	3.12
Y449	OH	Q42	NE2	H-bond	3.24
N487	OD1	Y83	OH	H-bond	2.25
Y489	OH	Y83	OH	H-bond	3.65
Q493	NE2	E35	OE2	H-bond	2.41
G496	O	K353	NZ	H-bond	2.54
Q498	NE2	Q42	OE1	H-bond	3.89
T500	OG1	Y41	OH	H-bond	2.43
T500	O	N330	ND2	H-bond	3.40
G502	N	K353	O	H-bond	3.13
Y505	OH	E37	OE2	H-bond	3.82
Y505	OH	R393	NH2	H-bond	3.74

Supplementary Table 4. Beta S-ACE2 structure revealed RBD-ACE2 interactions.

RBD from SARS-CoV-2 S		ACE2		Interaction	Distance (Å)
Residue	Atom	Residue	Atom		
Y449	OH	D38	OD2	H-bond	3.78
N487	OD1	Y83	OH	H-bond	2.24
Y489	OH	Y83	OH	H-bond	3.74
F490	O	K31	NZ	H-bond	3.89
Q493	OE1	K31	NZ	H-bond	2.40
Q493	NE2	E35	OE2	H-bond	2.38
T500	OG1	Y41	OH	H-bond	2.26
T500	O	N330	ND2	H-bond	2.90
Y501	OH	K353	NZ	H-bond	3.67
Y505	OH	R393	NH2	H-bond	3.49

Supplementary Table 5. Primers used in this study

Primer name	Primer sequence
Sp-F	TAGCGTTTAAACTTAAGCTTATGTTCGTGTTTCTGGTGCT
S-HIS-R	TTTAAACGGGCCCTCTAGACTCGAG
SP-686F	ACTCCCCAGGCTCCGCATCTTCTGTGGCAAGCCAGTCCATC
SP-685R	AGATGCGGAGCCTGGGGAGTTTGTCTGGGTC
SP-988-F	GCTGGACCCACCTGAGGCAGAGGTGCAGATCGAC
SP-987-R	CCTCAGGTGGGTCCAGCCGGCTCAGGATAT
D614G-R	GCACCTCGGTACAGTTCACGCCCTGATACAGCACGGCCACC
D614G-F	GGTGGCCGTGCTGTATCAGGGCGTGAACTGTACCGAGGTGC
E154K-F	TTGGATGAAGAGCGAGTTTCGCGTGTATTCCT
E154K-R	ACTCGCTCTTCATCCAAGACTTATTGTTC
L452R-F	CAATTATAGGTACCGGCTGTTTAGAAAGTCTAACC
L452R-R	GCCGGTACCTATAATTGTAGTTGCCGCCCACT
E484Q-F	CGTGCAGGGCTTTAACTGTTATTTCCCTCTGC
E484Q-R	AGTTAAAGCCCTGCACGCCATTG
P681R-F	CAGACCCAGACAAACTCCCGAGGCTCCGCATCTTCTGTGGC
P681R-R	GCCACAGAAGATGCGGAGCCTCGGGAGTTTGTCTGGGTCTG
L18-F	TGAGCTCCCAGTGCGTGAATTTTACCACAAGGACCCAGCTG
L18F-R	CAGCTGGGTCCTTGTGGTAAAATTCACGCACTGGGAGCTCA
D80A-F	CCAATGGCACAAAGAGGTTCGCCAACCCAGTGCTGCCC
D80A-R	GGGCAGCACTGGGTTGGCGAACCTCTTTGTGCCATTGG
D215G-F	CCCAATCAATCTGGTGAGAGGCCTGCCACAGGGCTTCTC
D215G-R	GAGAAGCCCTGTGGCAGGCCTCTCACCAGATTGATTGGG
242-246-F	CATCACCCGGTTTCAGACACTGCACATCAGCTACCTGACACC
242-246-R	GGTGTCAGGTAGCTGATGTGCAGTGTCTGAAACCGGGTGATG
K417N-F	GCACCAGGACAGACAGGCAACATCGCAGACTA
K417N-R	TAGTCTGCGATGTTGCCTGTCTGTCCTGGTGC
E484K-F	CCCTGCAATGGCGTGAAGGGCTTTAACT
E484K-R	AGTTAAAGCCCTTCACGCCATTGCAGGG
N501Y-F	CTACGGCTTCCAGCCAACATATGGCGTGGGCTATCAGCCCTA
N501Y-R	TAGGGCTGATAGCCCACGCCATATGTTGGCTGGAAGCCGTAG
A701V-F	TACCATGAGCCTGGGCGTCGAGAACTCCGTGGCCTAC
A701V-R	GTAGGCCACGGAGTTCTCGACGCCCAGGCTCATGGTA
ACE2-F	ACCGGGACCGATCCAGCCTCCGGACGCGGCCGC
ACE2-R	GTTGATTGTCGACTCTAGAATCAGTGATGATGGTGATGGTG GTGATGGTGGTCTGCATATGGACTCCAG
