# Supplementary material for: Conformational dynamics of the Beta and Kappa SARS-CoV-2 spike proteins and their complexes with ACE2 receptor revealed by cryo-EM
Source: Nat Commun. 2021 Dec 20;12:7345. doi: 10.1038/s41467-021-27350-0 (PMC8688474; doi:10.1038/s41467-021-27350-0)
Supplement: Supplementary file 3 — Description of Additional Supplementary Files [file 41467_2021_27350_MOESM3_ESM.pdf]

Description of Additional Supplementary Files

Supplementary Movie 1. A representative motion of the Kappa S-open dataset revealed by 3DVA.

Supplementary Movie 2. A representative 3DVA motion of the Beta S-open dataset.

Supplementary Movie 3. 3DVA motion mode 1 of the Kappa S-ACE2 complex.

Supplementary Movie 4. 3DVA motion mode 2 of the Kappa S-ACE2 complex.

Supplementary Movie 5. A representative 3DVA motion of the Beta S-ACE2 complex.
